# Deletion and Gene Expression Analyses Define the Paxilline Biosynthetic Gene Cluster in *Penicillium paxilli*

**DOI:** 10.3390/toxins5081422

**Published:** 2013-08-14

**Authors:** Barry Scott, Carolyn A. Young, Sanjay Saikia, Lisa K. McMillan, Brendon J. Monahan, Albert Koulman, Jonathan Astin, Carla J. Eaton, Andrea Bryant, Ruth E. Wrenn, Sarah C. Finch, Brian A. Tapper, Emily J. Parker, Geoffrey B. Jameson

**Affiliations:** 1 Institute of Fundamental Sciences, Massey University, Private Bag 11 222, Palmerston North 4442, New Zealand; E-Mails: cayoung@noble.org (C.A.Y.); ssaikia@msl.ubc.ca (S.S.) lisa.stanbra@scionresearch.com (L.K.M.); brendon.monahan@csiro.au (B.J.M.); j.astin@auckland.ac.nz (J.A.); c.j.eaton@massey.ac.nz (C.J.E.); akbryant@xtra.co.nz (A.B.); majoruth@hotmail.com (R.E.W.); emily.parker@canterbury.ac.nz (E.J.P.); g.b.jameson@massey.ac.nz (G.B.J.); 2 The Samuel Roberts Noble Foundation, Ardmore, OK 73401, USA; 3 AgResearch, Grasslands Research Centre, Private Bag 11 008, Palmerston North 4442, New Zealand; E-Mails: albert.koulman@mrc-hnr.cam.ac.uk (A.K.); brian.tapper@agresearch.co.nz (B.A.T.); 4 AgResearch, Ruakura Research Centre, East Street, Private Bag 3123, Hamilton 3214, New Zealand; E-Mail: sarah.finch@agresearch.co.nz

**Keywords:** indole-diterpene, paxilline, prenylation

## Abstract

The indole-diterpene paxilline is an abundant secondary metabolite synthesized by *Penicillium paxilli*. In total, 21 genes have been identified at the *PAX* locus of which six have been previously confirmed to have a functional role in paxilline biosynthesis. A combination of bioinformatics, gene expression and targeted gene replacement analyses were used to define the boundaries of the *PAX* gene cluster. Targeted gene replacement identified seven genes, *paxG*, *paxA*, *paxM*, *paxB*, *paxC*, *paxP* and *paxQ* that were all required for paxilline production, with one additional gene, *paxD*, required for regular prenylation of the indole ring post paxilline synthesis. The two putative transcription factors, *PP104* and *PP105*, were not co-regulated with the *pax* genes and based on targeted gene replacement, including the double knockout, did not have a role in paxilline production. The relationship of indole dimethylallyl transferases involved in prenylation of indole-diterpenes such as paxilline or lolitrem B, can be found as two disparate clades, not supported by prenylation type (e.g., regular or reverse). This paper provides insight into the *P. paxilli* indole-diterpene locus and reviews the recent advances identified in paxilline biosynthesis.

## 1. Introduction

Paxilline is a member of a large and structurally diverse group of indole-diterpene secondary metabolites, many of which are potent tremorgenic mammalian mycotoxins, synthesized by filamentous fungi [1]. These metabolites have a common structural core comprised of a cyclic diterpene skeleton derived from geranylgeranyl diphosphate (GGPP) and an indole group that is proposed to be derived from indole-3-glycerol phosphate, a precursor of tryptophan [2,3]. Paspaline is proposed to be the first stable intermediate from which many of the other metabolites of this class are derived [4]. Further chemical elaboration of paspaline is proposed to occur by additional prenylations, different patterns of ring substitutions and different ring stereochemistry [5].

Understanding fungal indole-diterpene biosynthesis has progressed considerably in recent years principally through research on paxilline biosynthesis in *Penicillium paxilli*. This is an ideal organism for studying indole-diterpene biosynthesis because it grows rapidly, produces large quantities of paxilline in submerged culture and is readily amenable to genetic manipulation [6,7]. Using a combination of plasmid insertional mutagenesis and chromosome walking, a cluster of genes was isolated and shown to be required for paxilline biosynthesis [8]. Gene disruption and chemical complementation experiments have shown that *paxG*, *paxP* and *paxQ* are required for paxilline biosynthesis [8,9,10]. 

**Figure 1:**
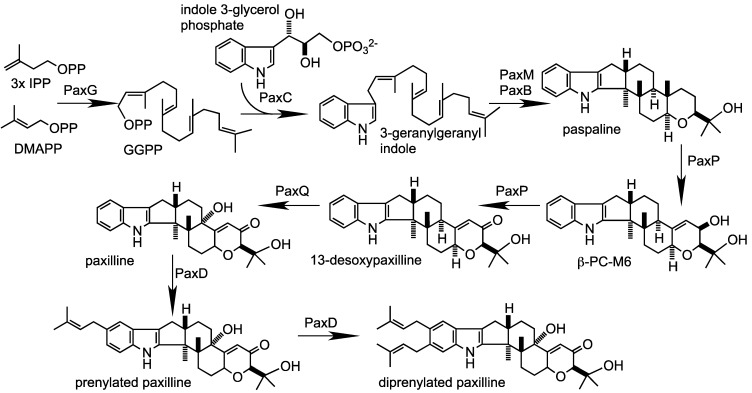
Proposed pathway for biosynthesis of paxilline and post-paxilline derivatives in *P. paxilli* based on experiments described here and in the recent work of Tagami *et al*. [12] and Liu *et al.* [13].

PaxG, a geranylgeranyl diphosphate (GGPP) synthase [11], is proposed to catalyze the first step in paxilline biosynthesis (Figure 1). Targeted deletion of *paxG* resulted in mutant strains that were completely blocked for indole-diterpene biosynthesis [8,11]. Using a *P. paxilli* mutant deleted for the entire *pax* gene cluster we were able to show by gene reconstitution experiments that just four genes, *paxG*, *paxM*, *paxB* and *paxC* were necessary and sufficient for paspaline biosynthesis [4]. Based on this study we proposed a biosynthetic scheme for paspaline biosynthesis involving condensation of indole-3-glycerol phosphate with GGPP to form 3-geranylgeranylindole (3-GGI), followed by epoxidation and cyclization of this intermediate to form paspaline (Figure 1). This scheme has recently been experimentally validated by reconstituting paspaline biosynthesis in the heterologous host *Aspergillus oryzae* [12]. Stepwise introduction of *paxG*, *paxC*, *paxM* and *paxB* into *A. oryzae*, combined with *in vitro* protein expression studies, demonstrated that PaxC is a prenyl transferase required for formation of 3-GGI and that PaxM and PaxB catalyze the stepwise epoxidation and cyclization of 3-GGI to paspaline [12]. Two cytochrome P450 monooxygenases, PaxP and PaxQ are involved in the later steps of the pathway in which paspaline is converted to paxilline [9,10]. While deletion mutants of *paxP* and *paxQ* were blocked for paxilline biosynthesis, they accumulated paspaline and 13-desoxypaxilline, respectively, confirming that both genes were required for paxilline biosynthesis and that paspaline and 13-desoxypaxilline were the most likely substrates for the corresponding enzymes [9]. This was confirmed by feeding these compounds to strains lacking the *pax* cluster but containing ectopically integrated copies of *paxP* and *paxQ* [10]. Transformants containing *paxP* converted paspaline into 13-desoxypaxilline as the major product and β-PC-M6 as the minor product. *paxQ*-containing transformants converted 13-desoxypaxilline into paxilline. These results confirmed that paspaline, β-PC-M6 and 13-desoxypaxilline are paxilline intermediates and that paspaline and β-PC-M6 are substrates for PaxP, and 13-desoxypaxilline is a substrate for PaxQ [10]. Stepwise introduction of the *pax* genes into *A. oryzae* showed that addition of *paxG-C-M-B-P-Q* was sufficient to reconstitute the machinery for paxilline biosynthesis [12].

Here we present a complete functional analysis of the *PAX* gene cluster locus. Using a combination of bioinformatics, gene expression and multiple targeted gene replacement analyses, we have demarcated the boundaries of the gene cluster and defined a set of seven genes required for paxilline biosynthesis in *P. paxilli*, plus one additional gene needed for paxilline prenylation. Collectively, the data presented here along with previously published results by us and others establish the *P. paxilli pax* gene cluster as a model system for understanding indole-diterpene biosynthetic pathways. 

## 2. Results and Discussion

Our first reported annotation of the *PAX* locus predicted the involvement of 17 genes in the biosynthesis of paxilline, with the *paxN* and *paxO* boundaries flanked by genes encoding a putative lipase and an arabinase, respectively [8]. A re-analysis of the DNA sequence at this locus identified a total of 21 putative genes, reannotated as *PP101* (*paxN*) to *PP121* (*paxO*). The four new putative genes identified were predicted to encode a hypothetical protein (PP103), an acetyl transferase (PP109) and two integral membrane associated proteins (PaxA/PP114 and PaxB/PP116). A summary of the putative functions of all 21 genes is summarized in Table 1.

**Table 1:** Putative functions of genes encoded at the *PAX* locus.

Gene/ ORF	Exon #	Predicted product size (aa)	Predicted function		Top BlastP hit			
			Description	InterProScan	Protein	Organism	*E*-value (%ID)	Accession/Ref
*PP101*	2	402	FAD-dependent oxidoreductase	IPR002938 IPR003042	Pc16g05940	*P. chrysogenum*	0.0 (78)	CAP93264
*PP102*	3	508	β-1,3- glucanosyltransferase	IPR004886 IPR012946 IPR013781 IPR013781		*A. oryzae*	0.0 (66)	BAE66482
*PP103*	3	103	Unknown	IPR018809		*P. digitatum*	3 × 10^−47^ (74)	EKV06610
*PP104*	2	477	Zn(II)_2_Cys_6_ transcription factor	IPR001138		*N. fischeri*	3 × 10^−81^ (34)	EAW19936
*PP105*	7	684	Zn(II)_2_Cys_6_ transcription factor	IPR001138 IPR007219		*A. oryzae*	0.0 (51)	XP_003189175
*PP106*	1	345	Unknown	IPR011042	Pc16g00180	*P. chrysogenum*	5 × 10^−164^ (66)	CAP92688
*PP107*	4	385	NADH oxidoreductase	IPR001327 IPR013027 IPR023753	Pc16g00160	*P. chrysogenum*	0.0 (65)	CAP92686
*PP108*	1	543	Capsule associated protein	IPR006598	CAP1	*Metarhizium anisopliae*	0.0 (55)	EFY96463
*PP109*	1	175	Acetyltransferase	IPR000182 IPR016181		*M. anisopliae*	3 × 10^−80^ (68)	EFY95041
*PP110*	3	818	Unknown	No hits		*A. niger*	0.0 (44)	EHA28514
*PP111*	3	478	Transporter (MFS)	IPR005828 IPR016196 IPR020846		*A. oryzae*	0.0 (75)	BAE63453
*PP112*	1	291	DUF829-Conserved protein family of unknown function	IPR008547	Pc13g04190	*P. chrysogenum*	5 × 10^−158^ (71)	CAP91488
*paxG* (*PP113*)	4	371	Geranylgeranyl diphosphate synthase	IPR000092 IPR008949 IPR017446	Pc20g01860	*P. chrysogenum*	1 × 10^-162^ (64)	CAP85515
*paxA* (*PP114*)	2	356	Integral membrane protein	No hits	AtmA	*A. flavus*	4 × 10^−462^ (33)	CAP53940/ [14]
*paxM* (*PP115*)	3	477	FAD-dependent monooxygenase	IPR002938 IPR003042	Pc20g01850	*P. chrysogenum*	0.0 (60)	CAP85514
*paxB* (*PP116*)	2	243	Integral membrane protein	No hits	AtmB	*A. flavus*	1 × 10^−103^ (62)	CAP53939/ [14]
*paxC* (*PP117*)	3	317	Prenyl transferase	IPR000092 IPR008949 IPR017446	Pc20g01840	*P. chrysogenum*	3 × 10^−162^ (69)	CAP85513
*paxP* (*PP118*)	6	515	Cytochrome P450 monooxygenase	IPR001128 IPR002403		*A. oryzae*	0.0 (64)	EIT78616
*paxQ* (*PP119*)	10	512	Cytochrome P450 monooxygenase	IPR001128 IPR002401 IPR017972	AtmQ	*A. flavus*	0.0 (60)	CAP53938/ [14]
*paxD (PP120)*	2	438	Indole dimethylallyl transferase	IPR012148 IPR017795	AtmD	*A. flavus*	2 × 10^−74^ (35) 5 × 10^−74^ (35)	EED52847 CAP53937/ [13,14]
*PP121*	4	418	FAD-binding oxidoreductase	IPR006094 IPR016166 IPR016167 IPR016169	W97_07461	*Coniosporium apollinis*	9 × 10^−88^ (34)	EON68203
*PP122*	3	306	Arabinase/Xylanase	IPR006710 IPR023296	Pc12g01330	*P. chrysogenum*	2 × 10^−42^ (38)	CAP79760

**Figure 2:**
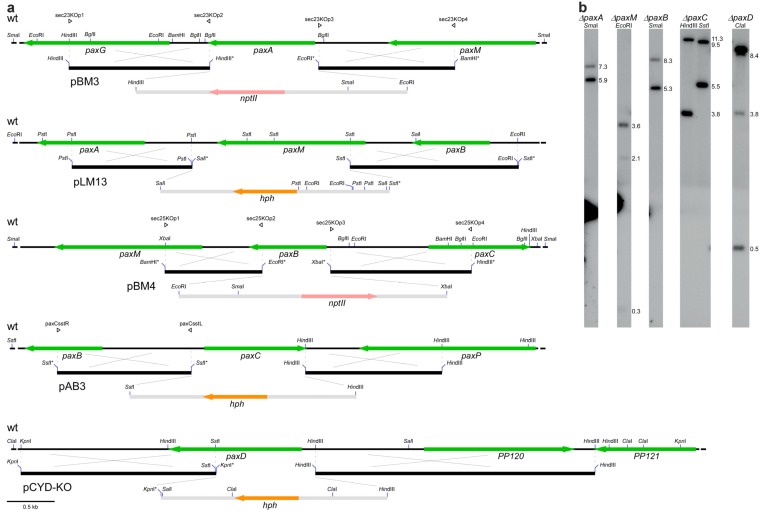
Paxilline biosynthesis gene replacements. (**a**) Physical maps of *P. paxilli* wild-type genomic region, linear replacement construct and mutant allele for each of *paxA* (*PP114*), *paxM* (*PP115*), *paxB* (*PP116*), *paxC* (*PP117*) and *paxD* (*PP120*); (**b**) Autoradiographs of Southern blots of 1 μg genomic digest of *P. paxilli* wild-type and mutant alleles, probed with [^32^P] dCTP-labeled replacement construct for each gene.

**Figure 3:**
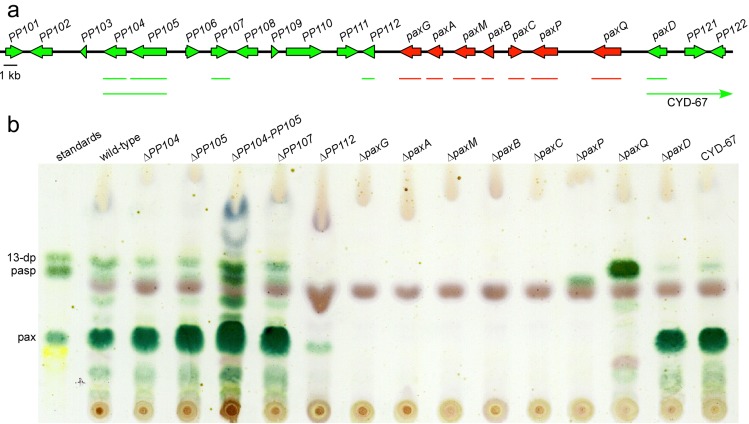
Deletion analysis of the paxilline biosynthesis gene cluster. (**a**) The *P. paxilli PAX* locus showing the organization of genes and ORFs. *Closed* arrows indicate the direction of gene/ORF transcription. The genes shown to be involved in paxilline and prenylated paxilline biosynthesis are designated as *pax* and the other predicted genes as *PP* (*Penicillium paxilli*). The *thin* red or green lines under the *PAX* locus indicate the deleted region for each gene or as an arrow in the case of the mutant CYD-67 that extends beyond the genomic region shown. Color scheme depicts role in paxilline biosynthesis based on gene deletion analysis: *red*—known role in paxilline biosynthesis; *green*—no role in paxilline biosynthesis, but *paxD* has a role in post-paxilline biosynthesis; (**b**) Normal phase TLC analysis for paxilline production in the *P. paxilli* strains deleted for the genes/ORFs mentioned in panel A. For paxilline extraction, mycelium was harvested 6 days after inoculation. Abbreviations: 13-dp, 13-desoxypaxilline; pasp, paspaline; pax, paxilline.

To define the core cluster of genes required for paxilline biosynthesis a set of targeted gene deletion mutations were generated at the *PAX* locus (Figure 2). PCR-generated linear fragments of the gene replacement constructs were recombined into the genome of *P. paxilli*. PCR screening of hygromycin or geneticin resistant transformants identified putative replacements. Southern blot analysis was used to identify transformants containing a targeted gene replacement (Figure 2). These transformants were analyzed by normal phase TLC for their ability to synthesize paspaline, 13-desoxypaxilline and paxilline (Figure 3). This analysis showed that Δ*paxG* [8,11], Δ*paxA*, Δ*paxM*, Δ*paxB* and Δ*paxC* mutants were unable to synthesize paxilline or any other indole-diterpene intermediates found in *P. paxilli* wild-type. The absence of any identifiable indole-diterpene compound in these extracts was confirmed by reverse phase HPLC analysis. As previously shown, Δ*paxP* and Δ*paxQ* mutants accumulate paspaline and 13-desoxypaxilline respectively [9]. Deletions of *PP104* and *PP105*, encoding putative transcription factors with Zn(II)_2_Cys_6_ binuclear cluster DNA-binding motifs, *PP107* (encoding a putative dehydrogenase), *PP112* (encoding a conserved hypothetical protein) and *paxD* (=*PP120*; encoding a putative indole dimethylallyl transferase) all accumulated paxilline and the other indole-diterpene intermediates found in *P. paxilli* wild-type. While the amount of paxilline present in the Δ*PP112* sample is low (Figure 3), independent TLC analyses confirmed this mutant did synthesize paxilline at levels comparable to the other mutants not involved in paxilline biosynthesis. The *PP104*-*PP105* double mutant also had the same phenotype as wild-type, as did CYD-67, a deletion of *paxD* that extends through *PP121* to an undefined point beyond both genes. This deletion analysis defines a set of 7 genes, *paxG* through to *paxQ*, that are required for paxilline biosynthesis.

**Figure 4:**
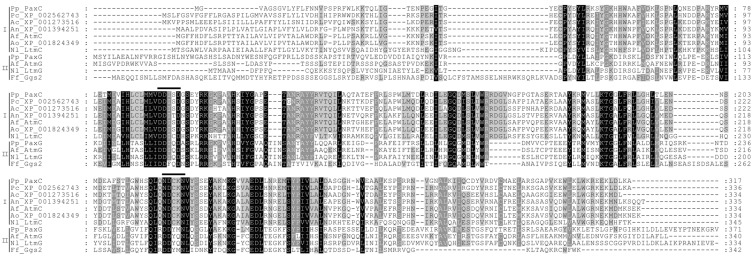
Alignment of predicted amino acid sequences for PaxC and related prenyltransferases. Numbers indicate the position of the last amino acid residue displayed. Sequences are grouped into I (PaxC-related, prenyl transferases) and II (geranylgeranyl diphosphate synthases). The aspartate-rich motifs are indicated by *bold* lines above the sequences. Sequences include genes from *Penicillium paxilli* (Pp_PaxC, AAK11529 and Pp_PaxG, AAK11531), *P. chrysogenum* (Pc_XP_002562743), *Aspergillus clavatus* (Ac_XP_001273516), *A. niger* (An_XP_001394251), *A. flavus* (Af_AtmC AAT65718 and Af_AtmG AAT65717), *A. oryzae* (Ao_XP_001824349), *Neotyphodium lolii* (Nl_LtmC, ABF20225.1 and Nl_LtmG, AAW88510) and *Fusarium fujikuroi* (Ff_Ggs2, CAA75568.1).

PaxC is predicted to be a prenyl transferase as it contains the five conserved domains found in other prenyl transferases [15] (Figure 4), including PaxG, which has recently been shown to be a functional GGPP synthase [11]. This superfamily of enzymes is characterized by the presence of two aspartate-rich motifs, DDXXD and DDXXN/D, located in Domains II and V, respectively, that are important for allylic substrate binding and catalysis. While the first aspartate-rich motif (DDISD) in PaxC conforms to this consensus, the second (NDXXN) does not suggesting PaxC has a novel function. The recent work by Tagami *et al.* [12] demonstrates that PaxC is a prenyl transferase that catalyzes the condensation of indole-3-glycerol phosphate with GGPP to form 3-GGI [12] (Figure 1).

**Figure 5:**
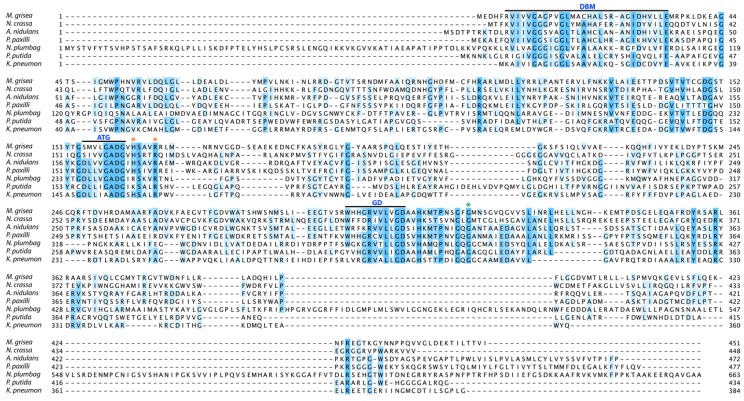
Alignment of amino acid sequences for PaxM and related FAD-dependent monooxygenases. Sequences were aligned using ClustalW and Jalview. The conserved dinucleotide binding motif (DBM), as well as the ATG, GD and G-helix (*) motifs found in functionally characterized FAD-dependent monooxygenases are highlighted. Sequences include: *P. paxilli* FAD-dependent monooxygenase (PaxM, AAK11530.1), *A. nidulans* hypothetical (ANID_11206.1), *N. crassa* fruiting body maturation protein, Fbm-1 (NCU02925.7), *M. oryzae* hypothetical (MGG_02256.6), *Nicotiana plumbaginifolia* zeaxanthin epoxidase (X95732.1) *Pseudomonas putida* salicylate hydroxylase, NahG (AAA25897.1) and *Klebsiella pneumoniae* FAD-dependent urate hydroxylase, UpxO (A6T923/3rp8).

PaxM is predicted to be an FAD-dependent monooxygenase containing a modified Rossman fold, as it contains the highly conserved dinucleotide binding motif (DBM), as well as the ATG, GD and G-helix motifs found in the functionally characterized salicylate hydroxylase (NahG) from *Pseudomonas putida* and zeaxanthin epoxidase from *Nicotiana plumbaginifolia* [16,17,18,19,20] (Figure 5). These same motifs are found in many closely related hypothetical proteins identified in the genomes of other filamentous fungi including fruiting body maturation (Fbm-1) from *Neurospora crassa* [21]. The top hits to PaxM were to structurally and (mostly) functionally characterized bacterial FAD-dependent, NAD(P)H-binding proteins including urate oxidase from *Klebsiella pneumoniae* (PDB ID: 3rp8; 22.3% identity) [22], 2,6-dihydroxypyridine 3-hydroxylase from *Arthrobacter nicotinovorans* (PDB ID: 2vou; 15.3% identity) [23], aklavinone-11-hydroxylase from *Streptomyces purpurascens* (PDB ID: 3ihg; 17.2% identity) [24] and putative FAD-containing monooxygenase from *Photorhabdus luminescens* subsp. *laumondii* TTO1 (PDB ID: 4hb9; 18.6% identity). Reconstitution of paspaline biosynthesis in *A. oryzae* demonstrates that PaxM, together with PaxB (see below), is involved in two rounds of epoxidation/cyclization to first generate emindole SB then paspaline [12] (Figure 1). 

**Figure 6:**
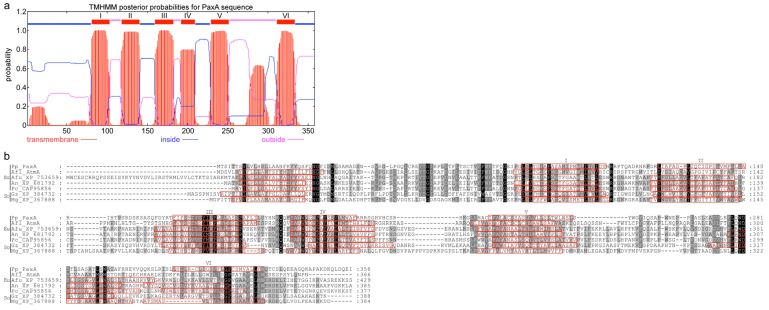
(**a**) Putative membrane topology of PaxA as determined by TMHMM; (**b**) Alignment of predicted amino acid sequences for PaxA and related proteins from representative fungi within the Eurotiomycetes (Eu) and Sordariomycetes (So). The predicted transmembrane helices, as determined by TMHMM, are indicated by *red boxes*. Numbers indicate the position of the last amino acid residue displayed. The predicted transmembrane helices in PaxA are labelled I-VI in both panel A and B. Sequences from *P. paxilli* (Pp_PaxA ADO29933), *A. flavus* (Afl_AtmA CAP53940.1), *A. fumigatus* (Afu_XP_753659), *A. nidulans* (An_XP_681792), *P. chrysogenum* (Pc_CAP95856), *Gibberella zeae* (Gz_XP_384732) and *Magnaporthe oryzae* (MGG_07792) are shown.

**Figure 7:**
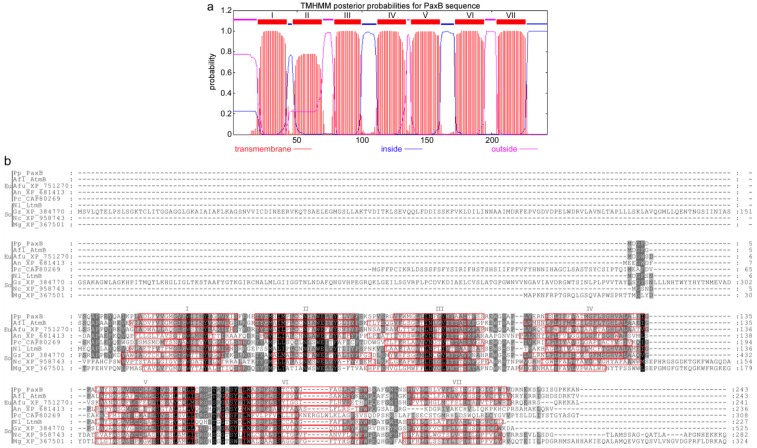
(**a**) Putative membrane topology of PaxB as determined by TMHMM; (**b**) Alignment of predicted amino acid sequences for PaxB and related proteins from representative fungi within the Eurotiomycetes (Eu) and Sordariomycetes (So). The predicted transmembrane helices, as determined by TMHMM, are indicated by *red boxes*. Numbers indicate the position of the last amino acid residue displayed. The predicted transmembrane helices in PaxB are labeled I-VII in both panel A and B. Sequences are from *P. paxilli* (Pp_PaxB, ADO29934), *A. flavus* (Afl_AtmB, CAP53939), *A. fumigatus* (Afu_XP_751270), *A. nidulans* (An_XP_681413), *P. chrysogenum* (Pc_CAP80269), *N. lolii* (Nl_LtmB, ABF20226), *G. zeae* (Gz_XP_384770), *N. crassa* (Nc_XP_958743) and *M. oryzae* (MGG_07412).

PaxA and PaxB appear to be a novel group of integral membrane proteins, containing 6 or 7 transmembrane domains (Figure 6, Figure 7). Despite their similarity in predicted secondary structure, they share very little sequence identity. They each contain a single intron, but the size (60 nt *versus* 87 nt) and location (345–404 and 519–605) of these introns is different. In addition, *paxA* utilizes a second 5' GT donor, upstream of the first (226–404; 170 nt intron), to generate an alternative mRNA isoform. Conceptual translation of this isoform generates a 77-, instead of 356-, amino acid polypeptide. The shorter (77 amino acid) predicted polypeptide contains no putative transmembrane domains. BLASTP analysis identified a number of closely related proteins in other fungal genomes but all are hypothetical conserved proteins. On the basis of their reconstitution experiments Tagami *et al.* [12] propose that PaxB is a novel indole-diterpene cyclase that works together with PaxM to convert 3-GGI to paspaline (Figure 1). However, the role of PaxA is unclear given reconstitution experiments in *P. paxilli* and *A. oryzae* demonstrated that *paxG-M-B-C* were required for the synthesis of paspaline [4,12], and in *A. oryzae* *paxG-M-B-C*-P-Q were sufficient for paxilline biosynthesis [12], yet the *paxA* deletion mutant was defective in paxilline biosynthesis and could be complemented by reintroduction of the wild-type *paxA*. Although, the functional role of PaxA is still unclear homologues of this gene are present in all *Penicillium* and *Aspergillus* indole-diterpene gene clusters identified to date [4,14]. Furthermore, a gene named *idtS* (*ltmS*) that encodes a structurally similar gene product to *paxA*, is found in indole-diterpene gene clusters from the Clavicipitaceae [25,26]. 

Given the mutual requirement of PaxB and PaxM to effect the conversion of 3-GGI to paspaline [12], it is of note that PaxM is predicted by TMHMM to have an approximately 25-residue *C*-terminal transmembrane helix with the *N*-terminal region in the cytosol. This *C*-terminal tag would facilitate co-location of PaxM with the integral membrane protein PaxB. Furthermore, the region of helix II predicted for PaxB is not predicted to be a transmembrane helix in several other sequences (Pc-CAP80269, Nl-LtmB and Mg_XP_367501; Figure 7). This region carries the conserved WExx(Y/F) motif in its middle. For Pc-CAP80269, Nl-LtmB and Mg_XP_367501 the *N*-terminal sequence preceding helix I is predicted to lie on the cytosolic side of the membrane, placing the conserved WExx(Y/F) extracellularly. At least one positively charged residue, as well as at least one histidine, is found on the intracellular loops between helices III and IV and between helices V and VI. The latter contain strongly conserved hydrophobic residues at their *N*- and *C*-termini, respectively. Finally, transmembrane predictor MEMSAT-SVM [27] suggests that PaxB has a propensity to form a pore. Based on all these observations we propose that PaxB may provide the proton(s) to break open the epoxide (the formation of which is mediated by PaxM) and orientate the 3-IGG in an internal pore so that the correct cyclization to paspaline takes place.

**Figure 8:**
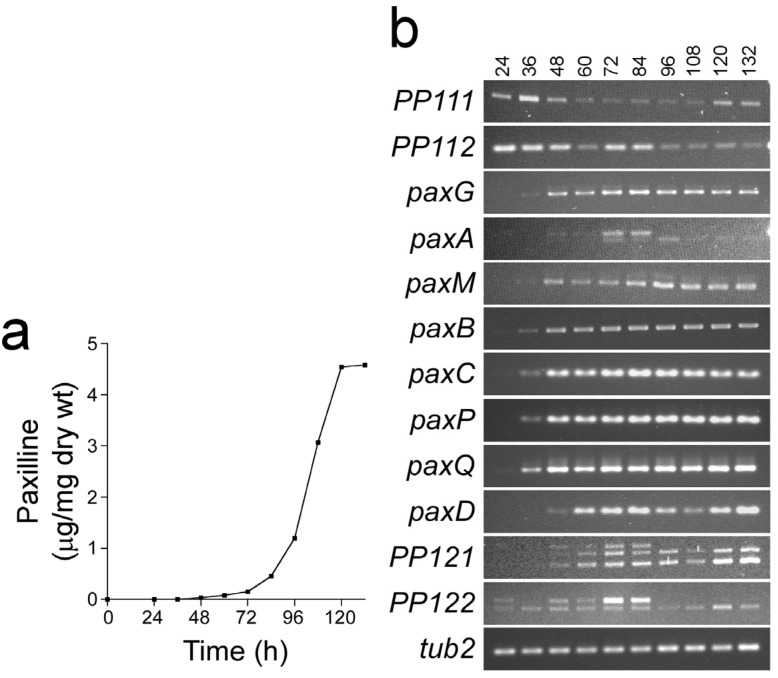
Coordinate expression of the *pax* genes is correlated with the onset of paxilline biosynthesis. (**a**) Time course of paxilline biosynthesis. For paxilline extraction, wild-type mycelium was harvested between 24 and 132 h of inoculation at 12 h intervals; (**b**) Expression analysis of *pax* genes. Total RNA was isolated from wild-type mycelium for each time point and used for cDNA synthesis. RT-PCR was performed with primers specific for each of the *pax* genes as well as the ORFs *PP111*, *PP112*, *PP121*, *PP122* and *tub2* (β-tubulin).

To further define the boundaries of the *pax* cluster, expression analysis was carried out on all proposed *pax* biosynthetic genes and on the genes immediately flanking the *pax* genes. This analysis showed that in addition to the 7 previously defined *pax* genes, *paxD* and *PP121* were also up-regulated with the onset of paxilline biosynthesis (Figure 8). The multiple bands observed in the *paxA*, *PP121* and *PP122* lanes are potentially products of incomplete or alternative splicing. In contrast to these samples the steady-state levels of β-tubulin, *PP111*, *PP112* and *PP122* are very similar across the time course of growth. These results suggest that *paxD* and possibly *PP121* are coordinately regulated with the 7 core *pax* biosynthetic genes. 

**Figure 9:**
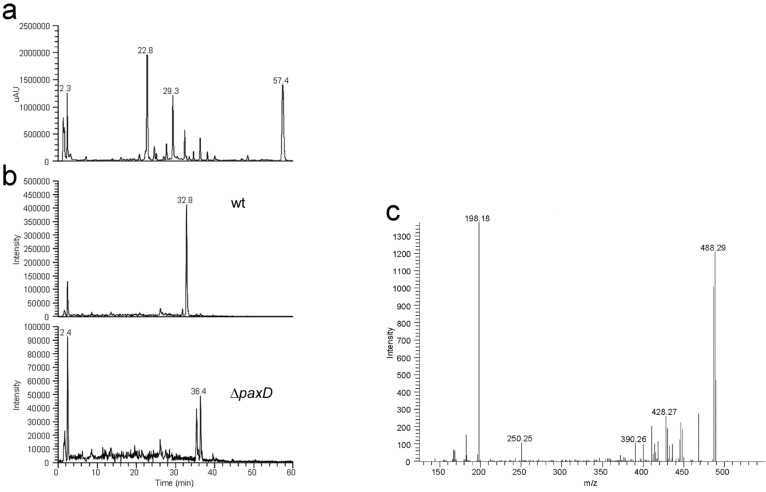
LC-MS/MS analysis of *P. paxilli paxD* deletion mutant. (**a**) UV trace at 275 nm of extract of *P. paxilli* wild-type; (**b**) Single ion extracted (504.3 *m/z*) chromatograms for wild-type and *paxD* deletion mutant; (**c**) Collision-induced fragmentation spectrum of the 504.3 *m/z* ion from wild-type (average of 6 mass spectra). Key ions are 488.3 (loss of CH_4_), 486.3 (loss of H_2_O), which is similar to paxilline fragmentation and 198.2 (prenylated indole). Based on these spectra, we assume that the prenylation occurs on the indole part of the molecule. However, the exact location of the prenyl group on the indole system remains to be elucidated.

The best characterized match to PaxD is AtmD from *Aspergillus flavus*, an indole dimethylallyl transferase that is predicted to catalyze the C4-reverse prenylation of paspalinine to form aflatrem [14,28] (Figure 1, Table 1). Therefore, a targeted disruption of *paxD* was made to determine whether there were any metabolite profile differences to wild-type that may be the result of additional prenylation steps. In screening the putative knockouts both a single replacement deletion (CYD-162) as well as an extended deletion of undefined length (CYD-67) of *paxD* were identified (Figure 2, Figure 3). As the TLC analysis of the *paxD* deletions showed the presence of paxilline, mass spectrometry (MS) analysis was used to compare the chemical phenotype of wild-type with the *paxD* deletion mutants. LC-MS/MS analysis identified a novel indole-diterpene at 32.8 min within the wild-type sample with a peak at *m/z* 504.3 that is absent in Δ*paxD* (Figure 9a,b). Based on MS analysis this compound is proposed to be an isoprenylated derivative of paxilline. MS2 fragmentation of this ion generated ions with peaks at *m/z* 488.3 (loss of CH_4_), 486.3 (loss of H_2_O) and 198.2 (prenylated indole) (Figure 9c). MS3 fragmentation of the ion at *m/z* 198.2 generated an ion with a peak at *m/z* 130 corresponding to the indole group (results not shown).

These results demonstrate that PaxD is able to catalyze the further addition of an isoprene unit to the basic paxilline structure (Figure 1), a result confirmed experimentally by Liu *et al.* [13] who demonstrated that PaxD purified from *E. coli* could catalyze the conversion of dimethylallyl diphosphate and paxilline *in vitro* to mono- (*m/z* of 504.3) and di-prenylated (*m/z* of 572.3) paxilline. Analysis of the ^1^H- and ^13^C-NMR spectra confirmed that the major product was 21,22-diprenylated paxilline [13]. The gene *PP121* is predicted to encode an oxidoreductase but this gene has still to be deleted to determine whether it also has a role in post-paxilline biosynthesis. However, the LC-MS/MS analysis was unable to detect differences between the Δ*paxD* (CYD-162) and the extended deletion mutant CYD-67, suggesting that if the *PP121* gene product has a role as part of this biosynthetic gene cluster, it would act post PaxD.

Unlike other prenyltransferases (e.g., PaxC and PaxG), the indole dimethylallyl transferases found in fungi do not contain the two aspartate-rich motifs, DDXXD and DDXXN/D, are generally more divergent [29,30,31,32], have broad indole derivative substrate specificity, yet only accept dimethylallyl diphosphate as the prenyl group donor [29]. The predicted active sites of two indole dimethylallyl transferases, CpaD (for α-cyclopiazonic acid) and FgaPT2 (first committed step in ergot alkaloid biosynthesis in *A. fumigatus*) have been characterized through mutagenesis and crystal structure, respectively [33,34]. CpaD and FgaPT2 both catalyze regular prenylation of the indole moiety at the C4 position and are found in the clade that contains the DmaW required for ergot alkaloid production (FgaPT2) or that catalyze a similar reaction (CpaD) [33,34,35,36]. Alignment of PaxD with these and other characterized dimethylallyl transferases shows some conservation across the sites proposed to be important for enzyme activity [33]. However, not all sites are conserved and these differences may explain enzymatic variation between substrates and resulting products where prenyl transfer occurs on different positions of indole moieties and depends on prenylation type. 

**Figure 10:**
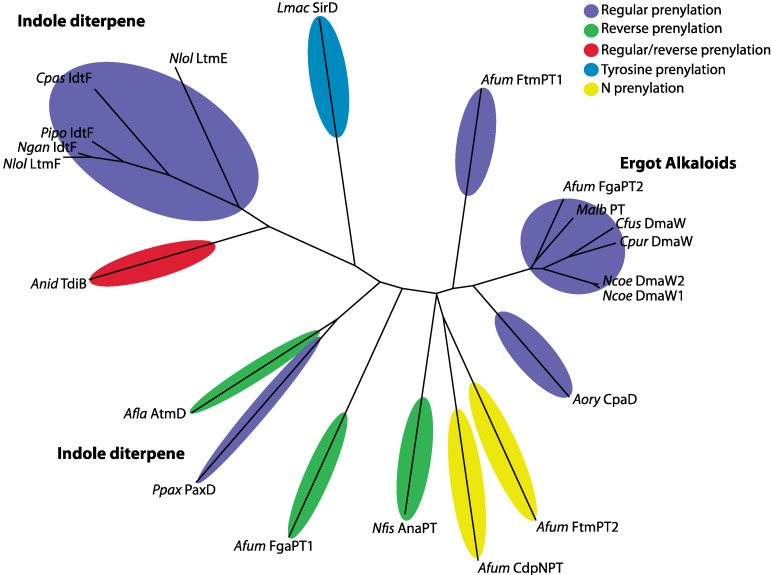
Unrooted tree of PaxD and related dimethylallyl transferases connected with enzymatic functions. The alignment consisted of 752 amino acids of which 265 sites were informative. Protein IDs with associated GenBank accession numbers are provided in Table A1 together with additional information on position and type of prenylation including name of the metabolite and reference.

To gain further insight into the evolution and functional relationship of PaxD and related indole dimethylallyl transferases, phylogenetic analysis of 21 related proteins, from 15 different species, of which 20 have known functions or predicted biosynthetic products, was carried out based on previous analyses of Liu *et al.* [37] (Figure 10). A phylogenetic tree based on the entire gene-coding region, of which 265 sites are informative, was used to potentially place functionality to PaxD. PaxD clustered closely with AtmD even though these two proteins share only 35% identity (Table 1) and have different modes of prenylation; regular for PaxD versus reverse for AtmD. The proteins within the ergot alkaloid clade, which includes DmaW, group very tightly together even though they have a broad taxonomic distribution [37]. In contrast, the dimethylallyl transferases involved in prenylation of indole-ditperpenes such as paxilline or lolitrem B, group into two very disparate clades (Figure 10). Although *P. paxilli* PaxD and *Claviceps paspali* IdtF are both able to prenylate the C5-position of an indole-diterpene resulting in prenylated paxilline and paspalitrem A, respectively, the sequences are quite divergent (sharing only 22% identity) and group in different clades. The two indole-diterpene clades represent members with different prenylation capabilities with both regular and reverse prenylation as well as prenylation of the diterpene moiety [26] (Table A1). Further analyses would be required to determine if the differences between these two clades simply represents phylogenetic distances between the species and whether there are implications for functional biochemical differences.

## 3. Experimental Section

### 3.1. Bacterial Strains and Plasmids

*Escherichia coli* strain XL1-Blue [38] was the host for routine cloning. The transformants of this host were grown on LB agar plates supplemented with ampicillin (100 μg/mL) for selection. Plasmids used in this study included pGEM^℘^-T Easy (Promega, Madison, WI, USA), pUC118 [39], pUChph [9], pCWHyg1 [8] and pII99 [40]. The bacterial strains used are listed in Table 2.

**Table 2:** Biological material.

Biological material	Targeted gene	Strain	Relevant characteristics	Reference
Fungal strains				
*Penicillium paxilli*				
PN2013			Wild-type; paxilline positive	[7]
PN2262	*PP104*	LMS-218	PN2013/∆PP104::P *trpC-hph*; Hyg^R^; paxilline positive	This study
PN2254	*PP105*	LMR-36	PN2013/∆PP105::P *trpC-hph*; Hyg^R^; paxilline positive	This study
PN2263	*PP104-PP105*	LMRS-173	PN2013/∆PP104, *∆*PP105*::PtrpC-hph*; Hyg^R^; paxilline positive	This study
PN2434	*PP107*	ABH-17	PN2013/∆PP107::P *trpC-nptII-TtrpC*; Gen^R^; paxilline positive	This study
PN2456	*PP112*	BMU-13	PN2013/∆PP112::P *trpC-nptII-TtrpC*; Gen^R^; paxilline positive	This study
PN2255	*paxG*	LMG-23	PN2013/∆ *paxG*::P*trpC-hph*; Hyg^R^; paxilline negative	[8]
PN2457	*paxA*	sec23-22	PN2013/∆ *paxA*::P*trpC-nptII-TtrpC*; Gen^R^; paxilline negative	This study
PN2257	*paxM*	LMM-100	PN2013/∆ *paxM*::P*glcA-hph-trpC*; Hyg^R^; paxilline negative	This study
PN2458	*paxB*	sec25-2	PN2013/∆ *paxB*::P*trpC-nptII-trpC*; Gen^R^; paxilline negative	This study
PN2290	*paxC*	ABC-83	PN2013/∆ *paxC*::P*trpC-hph*; Hyg^R^; paxilline negative	This study
PN2258	*paxP*	LMP-1	PN2013/∆ *paxP*::P*trpC-hph*; Hyg^R^; paspaline positive	[9]
PN2259	*paxQ*	LMQ-226	PN2013/∆ *paxQ*::P*trpC-hph*; Hyg^R^; 13-desoxypaxilline positive	[9]
PN2260	*paxD*	CYD-162	PN2013/∆ *paxD*::P*trpC-hph*; Hyg^R^; paxilline positive	This study
PN2261	*paxD-PP122+*	CYD-67	PN2013/∆ *paxD*::P*trpC-hph*; Hyg^R^; paxilline positive; extended deletion	This study
Plasmids				
pLM14 (pLMS-KO)	*PP104*		pUChph hygromycin resistance	This study
pLM15 (pLMR-KO)	*PP105*		pUChph hygromycin resistance	This study
pLM16 (pLMRS-KO)	*PP104-PP105*		pUChph hygromycin resistance	This study
pAB5 (pABH-KO)	*PP107*		pII99 geneticin resistance	This study
pBM2 (ppaxU-KO)	*PP112*	PN1942	pII99 geneticin resistance	This study
pBM3 (psec23-KO)	*paxA*	PN1944	pII99 geneticin resistance	This study
pLM13 (pLMM-KO)	*paxM*	PN1659	pCWHyg1 hygromycin resistance	This study
pBM4 (psec25-KO)	*paxB*	PN1946	pII99 geneticin resistance	This study
pAB3 (ppaxC-KO)	*paxC*		pUChph hygromycin resistance	This study
pCY1 (pCYD-KO)	*paxD*	PN1642	pCWHyg1 hygromycin resistance	This study

### 3.2. Fungal Strains and Growth Conditions

Cultures of *P. paxilli* Bainier wild-type (PN2013 = ATCC 26601) and deletion derivatives were routinely grown in PD, CDYE or ACM medium at 22 °C/28 °C for 4–6 days as previously described [4,9]. Liquid cultures were started using an inoculum of 5 × 10^6^ spores per 25 mL of CDYE medium supplemented with trace elements [4]. Media were supplemented with hygromycin (100 μg/mL) or geneticin (150 μg/mL), where necessary. The fungal strains used are listed in Table 2.

### 3.3. Molecular Biology

Plasmid DNA was isolated and purified by alkaline lysis using a Bio-Rad Quantum Prep^℘^ Plasmid Mini-prep Kit (Bio-Rad, Berkeley, CA, USA). Genomic DNA was isolated using a modification of the method of Yoder (1988) [41] as described previously [4]. PCR conditions were as previously described [4,9,10], using primer sets listed in Table A2. DNA fragments and PCR products were purified using a QIAquick gel extraction and PCR purification kit (Qiagen, Hilden, Germany). DNA fragments were sequenced by the dideoxynucleotide chain-termination method [42] using Big-Dye (Version 3) chemistry (PerkinElmer Life Sciences, Waltham, MA, USA) with oligonucleotide primers (Sigma Genosys, St. Lois, MO, USA). Products were separated on an ABI Prism 377 sequencer (Perkin-Elmer Life Sciences). Total RNA was isolated from frozen mycelium using TRIzol^℘^ reagent (Invitrogen, Carlsbad, CA, USA) and treated with DNase (Invitrogen, Carlsbad, CA, USA), as described previously [10]. RT-PCR conditions were as previously described [4,9,10], except DNase-treated total RNA (80 ng) was converted to cDNA and amplified for just 27 cycles in a single reaction using Superscript III-RT enzyme (Invitrogen) according to the manufacturer’s instructions. Primers used to amplify each of the genes are summarized in Table A2.

### 3.4. Preparation of Deletion Constructs

Plasmid pLMS-KO (paxR2#8) (pLM14; *PP104* replacement) was constructed by sequentially ligating into pUChph, a 1.03 kb *Sst*I fragment 5' of PP104 and a 1.16 kb *Hin*dIII fragment 3' of PP104, sourced from PCR products amplified from *P. paxilli* genomic DNA with primer sets paxU1SstIL/pax66 and paxRU1HindIIIL/paxRU1HindIIIR, respectively. Plasmid pUChph was digested with *Sst*I and *Hin*dIII.

Plasmid pLMR-KO (pLM15; *PP105* replacement) was constructed by sequentially ligating into pUChph, a 1.1 kb *Bam*HI fragment 5' of PP105 and a 1.1 kb *Hin*dIII fragment 3' of PP105, sourced from PCR products amplified from *P. paxilli* genomic DNA, with primer sets paxRBamL/paxRBamR and KORH/KORHS respectively. Plasmid pUChph was digested with *Bam*HI and *Hin*dIII.

Plasmid pLMSR-KO (paxR1/R2#5) (pLM16; *PP104* & *PP105* replacement) was constructed by sequentially ligating into pUChph, a 1.1 kb *Bam*HI fragment 5' of PP105 and a 1.16 kb *Hin*dIII fragment 3' of PP104, sourced from PCR products amplified from *P. paxilli* genomic DNA, with primer sets paxRU1BamR/paxRU1BamL and paxRU1HindIIIL/paxRU1HindIIIR respectively. Plasmid pUChph was digested with *Bam*HI and *Hin*dIII.

Plasmid pAB40 (*PP107* replacement) was constructed by sequentially ligating into pII99 a 1.27 kb *Bgl*II/blunt-end fragment 3' of *paxH* and a 1.5 kb *Hin*dIII/*Xho*I fragment 5' of *paxH*, sourced from PCR products amplified from λCY42 [8] with primer sets pax175/pax183 and pax251Hind/pax186, respectively. Plasmid pII99 was digested with *Bgl*II/*Eco*RV and *Xho*I/*Hin*dIII.

Plasmid pBM2 (*PP112* replacement) was constructed by sequentially ligating into pII99, a 1.5 kb *Xba*I/*Hind*III fragment 5' of *PP112* and a 1.6 kb *Bam*HI/*Eco*RI fragment 3' of *paxA*, sourced from PCR products amplified from *P. paxilli* genomic DNA with primer sets paxUKOp3/paxUKOp4 and paxUKOp1/paxUKOp2. Plasmid pII99 was digested with *Xba*I/*Hind*III and *Bgl*II/*Eco*RI. 

Plasmid pBM3 (*paxA* replacement) was constructed by sequentially ligating into pII99, a 1.4 kb *Bam*HI/*Eco*RI fragment 5' of *paxA* and a 1.5 kb *Hind*III fragment 3' of *paxA*, sourced from PCR products amplified from *P. paxilli* genomic DNA, with primer sets sec23KOp4/sec23KOp3 and sec23KOp2/sec23KOp1, respectively. Plasmid pII99 was digested with *Bgl*II/*Eco*RI and *Hind*III. 

Plasmid pLMM-KO (pLM13; *paxM* replacement) was constructed by sequentially ligating into pUC118, a 1.3 kb *Pst*I fragment 3' of *paxM*, a 2.3 kb *Sal*I fragment, containing the *hph* gene, and a 1.8 kb *Sst*I fragment 5' of *paxM*, sourced from λCY46 [8], plasmid pCWHyg1 [8] and an *Sst*I digest of CY46-11 (pUC118 containing a 1.8 kb *Eco*RI fragment from λCY46).

Plasmid pBM4 (*paxB* replacement) was constructed by sequentially ligating into pII99, a 1 kb *Bam*HI/*Eco*RI fragment 3' of *paxB* and a 1.5 kb *Xba*I/*Hin*dIII fragment 5' of *pax*B, sourced from PCR products amplified from *P. paxilli* genomic DNA with primer sets sec25KOp1/sec25KOp2 and sec25KOp3/sec25KOp4. Plasmid pII99 was digested with *Bgl*II/*Eco*RI and *Xba*I*/Hind*III. 

Plasmid pAB3 (*paxC* replacement) was constructed by sequentially ligating into pUChph (McMillan 2003), a 1.4 kb *Hin*dIII fragment 3' of *paxC* from clone 56H-14, a *Hin*dIII pUC118 sub-clone of λCY56 [8], and a 1.4 kb *Sst*I fragment 5' of *paxC* from a PCR product amplified from *P. paxilli* genomic DNA with primer set paxCSstR/paxCSstL. 

Plasmid pCYD-KO (*paxD* replacement) was constructed in two steps. The first step involved a three-way ligation of *Hin*dIII/*Sal*I cut pUC118 with a 2.9 kb *Hin*dIII fragment 5' of *paxD* from clone CY56-19 (a 5-kb *Sst*I pUC118 sub-clone of λCY56) [8] and a 2.3 kb *Hin*dIII/*Sal*I fragment containing *hph* from pCWHyg1 [9]. The resulting plasmid was digested with *Kpn*I and ligated with a 2.2 kb *Kpn*I fragment 3' of *paxD* from clone 56-1, a 10.5 kb *Sst*I pUC118 sub-clone of λCY56 [8].

### 3.5. *Penicillium paxilli* Transformation and Screening

Protoplasts of PN2013 were prepared and transformed with PCR-amplified linear products of each of the replacement constructs as previously described [9], except protoplasts transformed with linear products of pBM2, pBM3 and pBM4 were plated on ACM medium supplemented with 0.8 M sucrose, rather than RG medium. Transformants were selected on medium supplemented with either hygromycin (100 μg/mL) or geneticin (150 μg/mL). The resulting stable transformants were maintained on either PD or ACM medium supplemented with either hygromycin or geneticin. 

Primary screening of transformants for targeted homologous recombination events was carried out using genomic DNA from conidia as template [4], and primer sets (see above) within, and external to, the gene fragment to be replaced. Putative replacement mutants identified by PCR screening were further analyzed by Southern blotting and hybridization, using methods previously described [6].

### 3.6. Indole-Diterpene Analysis

Indole-diterpenes were extracted from mycelium of *P. paxilli* in a 2:1 chloroform-methanol mixture and analyzed by normal phase TLC and reverse phase HPLC as previously described [4]. LC-MS/MS analysis was performed on a Thermo Finnigan Surveyor (Thermo Finnigan, San Jose, CA, USA) HPLC system as previously described [4]. Mass spectra were determined with a linear ion trap mass spectrometer (Thermo LTQ, Thermo Finnigan, San Jose, CA, USA) using electro spray ionization (ESI) in positive mode using parameters previously described [4].

### 3.7. Bioinformatic Analyses

Sequences were aligned using ClustalX or ClustalW [43] with sequences retrieved from the NCBI GenBank database or the Broad Institute. Multiple sequence alignments were edited using Jalview.

Putative function of proteins encoded by *pax* genes and protein domains were identified using InterProScan [44,45]. The predicted transmembrane topologies of PaxA and PaxB were determined using TMHMM version 2, which utilizes a hidden Markov model [46].

Given the low level of sequence identity to proteins of known function, pGenThreader [47] at the University College London website [27] was used to find structures whose pattern of secondary structure elements match those predicted for PaxM. This threading is based on the well-established premise that 2-D structures, for which reliable prediction algorithms exist [48], and resultant 3-D structures, are conserved even where sequence identity has lost significance. 

The phylogenetic relationships of PaxD and other known indole dimethylallyl transferases (accession numbers provided in Table A1) were determined with the program MAFFT version 7 [49,50]. Alignments were performed similarly to Liu *et al.* [37] with the following settings, FFT-NS-I, JTT200 scoring matrix with the gap opening penalty set to 1.0 and gap extension penalty at 0.0.

The *pax* gene sequences from *P. paxilli* are available in the GenBank database under accession number HM171111 (update to AF279808).

## 4. Conclusions

A cluster of seven genes—*paxG*, *paxA*, *paxM*, *paxB*, *paxC*, *paxP* and *paxQ*—is required for paxilline biosynthesis in *P. paxilli*. One additional gene, *paxD*, is required for a post-paxilline biosynthetic step resulting in prenylation of the indole group of paxilline. Together, these genes constitute the *pax* gene cluster with each gene deleted, functionally characterized, and shown to be transcriptionally co-regulated.

## Appendix

**Table A1:** Features of dimethylallyl transferases used in Figure 10.

Species	Isolate	Prenylation Type	Position	Protein	Compound	Reference
*Aspergillus flavus*	NRRL 6541	reverse	C4	AtmD	aflatrem (IDT)	[14]
*Aspergillus fumigatus*	Af293	regular	C2	FtmPT1	Brevianamide F	[51]
*Aspergillus fumigatus*	Af293	reverse	C2	FgaPT1	fumigaclavine C	[35]
*Aspergillus fumigatus*	Af293	regular	C4	FgaPT2	fumigaclavine C	[35]
*Aspergillus fumigatus*	Af293	N-PT-reverse	N1	CdpNPT	cyclic dipeptide *N*-prenylated	[52,53]
*Aspergillus fumigatus*	Af293	N-PT-regular	N1	FtmPT2	fumitremorgin B	[54,55]
*Aspergillus nidulans*	Un-specified	regular and reverse	Reg = quinone; Rev = C2	TdiB	terrequinone A	[56]
*Aspergillus oryzae*	RIB 40	regular	C4	CpaD	cyclopiazonic acid	[33,36]
*Claviceps fusiformis*	ATCC26245	regular	C4	DmaW	ergot alkaloid	[32]
*Claviceps paspali*	RRC-1481	regular	C5	IdtF	paspalitrem A (IDT)	[25]
*Claviceps purpurea*	P1	regular	C4	DmaW	ergot alkaloid	[30]
*Leptosphaeria maculans*	ICBN 18	Tyrosine-regular	O	SirD	sirodesmin	[57]
*Malbranchea aurantiaca*	RRC1813	regular	C4	MaPT	ergot alkaloid	[58]
*Neosartorya fischeri*	NRRL 181	reverse	C3	AnaPT	acetylaszonalenin	[59]
*Neotyphodium coenophialum*	ATCC90664	regular	C4	DmaW	ergot alkaloid	[60]
*Neotyphodium gansuense*	e7080	regular		IdtF		[25]
*Neotyphodium lolii*	Lp19	regular	ggpp moiety	LtmF	lolitrem B (IDT)	[61]
*Neotyphodium lolii*	Lp19	regular	C4, C5	LtmE	lolitrem B (IDT)	[61]
*Penicillium paxilli*	ATCC26601	regular (di)	C5, C6	PaxD	Indole-diterpene (IDT)	[8]
*Periglandula ipomoeae*	IasaF13	regular	ggpp moiety	IdtF	terpendole K (IDT)	[25]

**Table A2:** List of primers for replacement constructs and RT-PCR.

Gene	Primer 1	Sequence (5'–3') ^a^	Primer 2	Sequence (5'–3')	Size (kb)	Application
*PP104*	paxU1SstIL	CTTGTTGGCgaGCTcCATATGAC	pax66	CGCGATGGCGTACTGTAGAC	1.03	KO construct
*PP104*	paxRU1HindIIIL	TTTAGTAGAAGCTTGGCC	paxRU1HindIIIR	TCGTTGAAGcTTGCAGTA	1.16	KO construct
*PP105*	paxRBamL	ATTGACGgATCCCGATTATC	paxRBamR	GGATccGAGATGGGTGTATAC	1.1	KO construct
*PP105*	KORH	GGGGTATAaGcTTAACATAGAGCAG	KORHS	GTTACATGCTTCCATTTAAAGTTGGGAGCTGTC	1.1	KO construct
*PP104* & *PP105*	paxRU1BamR	CAACGTTGTGGATCCATTCGG	paxRU1BamL	CCCTATCGGGATGCAATTTTCAAAC	1.1	KO construct
*PP107*	pax251Hind	TGCAAGCTTCCGCTATAG	pax186	AGTCAACACCAAGACAGG	1.27	KO construct
*PP107*	pax175	TCGACGACTTCGACCAGA	pax183	ATGTCATCTTCCGCAATC	1.5	KO construct
*PP112*	paxUKOp3	ACGTTGCTAGTcTAGaTGGAAGC	paxUKOp4	AGTTCGTaAGCTTGATGTGTTG	1.5	KO construct
*PP112*	paxUKOp1	GTGATGGATcCCAAAATTCATTGG	paxUKOp2	GCAAGAaTTCAAATGCCTGGAAG	1.6	KO construct
*paxA*	sec23KOp3	TGGCCGAaTTCCGAGAATAGAGT	sec23KOp4	AAGAAATACGTGgATCCTGACAG	1.4	KO construct
*paxA*	sec23KOp2	GCTAAAGcTtAACAACTGGACCA	sec23KOp1	CTCGACAAGcTTAGAAAAGTCAC	1.5	KO construct
*paxB*	sec25KOp3/	CGTTGAATAGCTcTAGATTGAAGG	sec25KOp4	TGAGCCAAgcTTTGTGTAACTCG	1.0	KO construct
*paxB*	sec25KOp1/	GTGATgGATCCCAAAATTCATTGG	sec25KOp2	GCAAGaATTCAAATGCCTGGAAG	1.5	KO construct
*paxC*	paxCSstR	GTTGAGCTCAATCCACCAACGC	paxCSstL	GAGTGAGCTCTGCTTGGTAGGC	1.4	KO construct
*PP112*	paxU RT-F	TCGTCCTATCTCGCACCTTTC	paxU RT-R	AGAGTCTGTTCGGTTCGATGG	474	RT-PCR
*paxG*	paxG RT-F	ACACTGCATCCCTTCTTATCG	paxG RT-R	TATCGAGAAGCTCGGAGCTCT	528	RT-PCR
*paxA*	paxA RT-F	CAACCTTTCAGGGTGAGATTC	paxA RT-R	CAGATGAGCAAGCCAAGGCAA	489	RT-PCR
*paxM*	paxM RT-F	TCATCGATCAAAGGTTCGGTT	paxM RT-R	AACTCGACCGTAAGCTTGGAA	301	RT-PCR
*paxB*	paxB RT-F	GAACTGGTCTACTGTCTGGTC	paxB RT-R	ACGGTCGACGTACCAGAAACA	504	RT-PCR
*paxC*	paxC RT-F	ATGATGGTCGACGATATCTCC	paxC RT-R	CAATTGCGAATGCCAGCCAAG	385	RT-PCR
*paxP*	paxP RT-F	CCACCTACAAGACCAATGTCA	paxP RT-R	AAGCGAATTGATCATCGCATG	417	RT-PCR
*paxQ*	paxQ RT-F	CAGCCTTACAGAGAGATTCGT	paxQ RT-R	GATGTGCGACAACTCTTGCAC	562	RT-PCR
*paxD*	paxD RT-F	CAGTCTGGAGCTTATGCCATC	paxD RT-R	CGTCCTTGACGAATGCCTTGA	456	RT-PCR
*PP121*	paxO RT-F	GTGGCTGCTACTAAGCTGGTA	paxO RT-R	CACAGGAAGAAGCGATCTGGT	456	RT-PCR
*PP122*	Pax248	AGTTCGACAGCGCTTGGGAGA	Pax249	CAGTGGCTCCTTAACTCTCGT	592	RT-PCR
*tubA*	Tub2 RT-F	ACACTCCTGATCTCCAAGATC	Tub2 RT-R	GATGTCGTACAGAGCCTCGTT	258	RT-PCR

^a^ Nucleotides in lower case are mismatches to genomic sequence to introduce sites for restriction enzyme digestion.
